# Supplementary material for: Developing an Evidence-Based Nursing Culture in Nursing Homes: An Action Research Study
Source: Int J Environ Res Public Health. 2022 Feb 2;19(3):1733. doi: 10.3390/ijerph19031733 (PMC8835437; doi:10.3390/ijerph19031733)
Supplement: Supplementary file 1 [file ijerph-19-01733-s001.zip › ijerph-1563532-supplementary.pdf]

Table S1
The nine principles of the Practice Development approach

#1	*Person-centered and different types of evidence*<br>The goal is to develop an EBN culture in which different types of evidence – not limited to research evidence – are valued and used.
#2	*Micro-level supported by meso- and macro-levels*<br>The focus is on the micro-level (where care takes place), but it is important that this level be supported by management.
#3	*Learning on the spot*<br>Learning is part of working, and it's important to find/create learning opportunities.
#4	*Developing and applying evidence*<br>Knowledge is applied and enriched by sharing experiences, making action plans, and reflecting on practice.
#5	*Creativity combined with cognition*<br>Creativity (e.g., drawing, using picture cards, sculpting) and more analytical methods are used to stimulate nurses to think out of the box when looking for ways to improve practice.
#6	*Involvement of stakeholders*<br>Stakeholder involvement is important because it gives everyone a say and an opportunity to influence the process and results of the actions. Frequently used methods include visualizing the workplace culture, creating a shared vision, and evaluating with all stakeholders by using a Fourth Generation Evaluation (claims, concerns, and issues).
#7	*Tailored methods*<br>Tailored methods are used that align with the context of care and the stakeholders in the context.
#8	*Facilitating*<br>A facilitator is needed to support or facilitate the development of skills so the intended actions are carried out and the goals can be achieved.
#9	*Stakeholder involvement in evaluation*<br>Every relevant stakeholder will be asked for feedback because their view is important for the further development of practice.

Table S2

Example of a Tailored Fundamental Nursing Care Topic for Improvement

Topic	Oral Care
Problem in practice	A resident on one of the wards had bad breath and did not want to brush his teeth. The family experienced his bad breath as a problem, and the nursing team could not provide him with oral care because he did not want to cooperate.
Research question	The nursing team formulated these research questions: What is good oral care? How can we prevent bad breath or toothache? How do we provide good oral care to a resident who does not want to cooperate?
Assessment of daily practice	The nursing team mapped the oral care they provided, the wishes and routines of the residents, and the extent to which the residents could take care of their mouths and teeth by themselves. In addition, the nursing team gained insight into the residents' oral health and complaints. The team studied the national guideline and protocols about oral care, and they consulted a dentist.
Improvement	Based on all the collected information, the nursing team revised the residents' care plans. All residents received the right oral care according to protocol, like a regular check-up by the dentist and tooth brushing.
Conclusion	Residents should be encouraged to take good care of their mouth and teeth every day. All residents now have the right materials for doing so in their own rooms.
